# Severe Agranulocytosis and Thyroid Storm Triggered by Reinitiating Low-Dose Thiamazole: A Cautionary Case

**DOI:** 10.1210/jcemcr/luaf330

**Published:** 2026-02-04

**Authors:** Yuki Minamoto, Kenta Amemiya, Yui Yamashita, Yumiko Sasai, Keiko Yamagami, Naotetsu Kanamoto

**Affiliations:** Department of Endocrinology, Osaka City General Hospital, Osaka 534-0021, Japan; Department of Endocrinology, Osaka City General Hospital, Osaka 534-0021, Japan; Department of Diabetes and Endocrinology, Hyogo Prefectural Amagasaki General Medication Center, Hyogo 660-8550, Japan; Department of Endocrinology, Osaka City General Hospital, Osaka 534-0021, Japan; Department of Endocrinology, Osaka City General Hospital, Osaka 534-0021, Japan; Department of Endocrinology, Osaka City General Hospital, Osaka 534-0021, Japan; Department of Endocrinology, Osaka City General Hospital, Osaka 534-0021, Japan

**Keywords:** agranulocytosis, antithyroid drug, Graves disease, thyroid storm

## Abstract

Antithyroid drug (ATD)-induced agranulocytosis is a rare but potentially life-threatening adverse effect. We report the case of a 51-year-old man who developed agranulocytosis after reinitiating treatment with low-dose thiamazole, which was complicated by right buccal cellulitis, leading to thyroid storm. He was diagnosed with Graves disease 4 years earlier and treated with thiamazole; however, he discontinued the treatment on his own after 1 year. Due to the recurrence of Graves disease, 5 mg of thiamazole was reinitiated. Thirty-five days later, thyroid storm occurred, owing to right buccal cellulitis with thiamazole-induced agranulocytosis. Thiamazole can induce agranulocytosis, even at low doses and when reinitiated, regardless of prior tolerance. This case emphasizes that prior tolerance to thiamazole does not preclude the possibility of life-threatening adverse events upon reinitiation, even at minimal doses. Even in the absence of adverse effects during the initial course of ATD therapy, physicians should remain vigilant for ATD-related adverse effects when reinitiating treatment, even at low doses, particularly after a prolonged discontinuation period. Given the severity of agranulocytosis, patient education at the time of prescription is crucial for the early recognition of symptoms, such as fever or sore throat, which may enable timely diagnosis and appropriate intervention.

## Introduction

Antithyroid drug (ATD)-induced agranulocytosis is a rare but potentially life-threatening adverse effect that requires prompt recognition and intervention to achieve a favorable prognosis [1]. The incidence of agranulocytosis caused by thiamazole increases in a dose-dependent manner [2, 3]. Its pathogenesis is believed to involve both immune-mediated mechanisms and direct toxic effects on neutrophils [4]. Herein, we report the case of a 51-year-old man who developed agranulocytosis after reinitiating treatment with low-dose thiamazole, which was complicated by right buccal cellulitis leading to thyroid storm.

## Case Presentation

A 51-year-old man, who had no specific medical history, was diagnosed with Graves disease 4 years earlier. He was initially treated with 10 mg of thiamazole and later maintained at 5 mg without adverse effects; however, he discontinued treatment with thiamazole on his own 1 year later. At 3 years after discontinuation, he presented with facial and lower leg edema and was diagnosed with heart failure and atrial fibrillation during a previous hospitalization. Thyroid function tests demonstrated an undetectable thyroid-stimulating hormone (TSH) level (reference range: 0.50-5.00 μIU/mL [0.50-5.00 μIU/L]) and a free thyroxine (FT4) level of 2.1 ng/dL (27.0 pmol/L) (reference range: 0.9-1.7 ng/dL [11.6-21.9 pmol/L]). Thiamazole 5 mg was reinitiated for Graves disease, and the neutrophil count remained within the normal range, with the absolute neutrophil count monitored repeatedly over 10 inpatient days. Thirty-five days after the reinitiation of thiamazole, facial itching appeared, followed by fever, diarrhea, and loss of appetite the following day. Three days later, he visited a primary care physician. Agranulocytosis was suspected based on a neutrophil count of 80/μL (0.08 × 10^9^/L) (reference range at the previous hospital: 1500-5000/μL [1.5-5.0 × 10^9^/L]) and a C-reactive protein (CRP) level of 18.55 mg/dL (185.5 mg/L) (reference range: < 0.3 mg/dL [< 3 mg/L]); subsequently, he was transferred to our hospital. The patient had no family history of thyroid disorders. He had no prior medical history and was taking thiamazole (5 mg), bisoprolol (2.5 mg), enalapril (2.5 mg), spironolactone (25 mg), empagliflozin (10 mg), azosemide (30 mg), edoxaban (60 mg), and rabeprazole (10 mg), all of which were started concomitantly and taken once daily.

## Diagnostic Assessment

**Figure 1.**
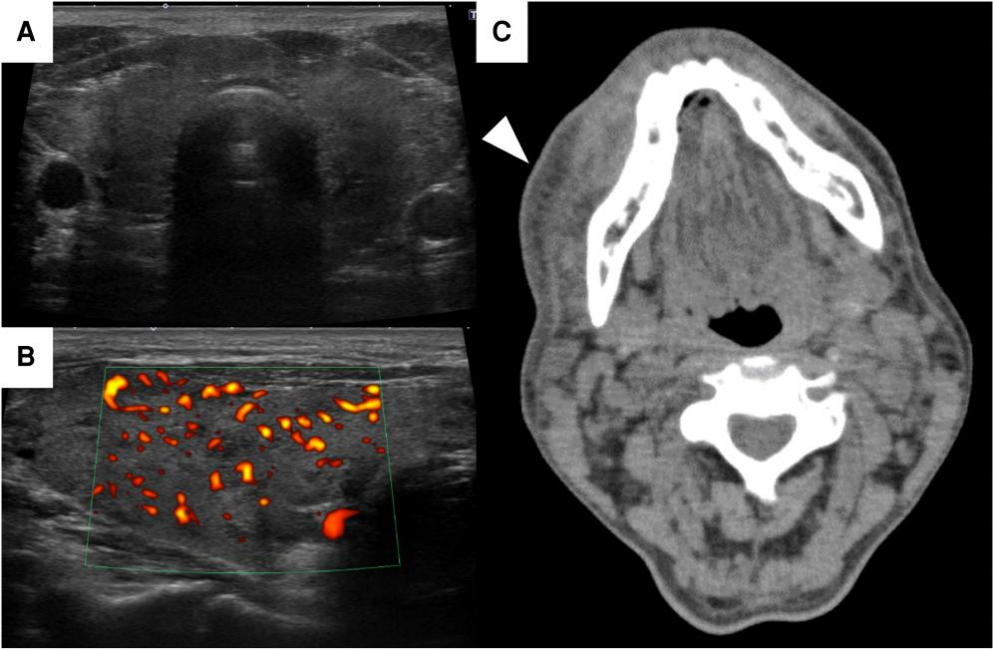
Transverse view (A) and power Doppler examination (B) of the thyroid ultrasonography. The thyroid appears enlarged and heterogeneous with increased vascularity on power Doppler imaging. Computed tomography of the head and neck (C). Soft tissue opacity is observed in the right buccal region (*arrowhead*).

On examination in the emergency department, he exhibited a mildly impaired level of consciousness, a fever of 41.3 °C, and diarrhea. Atrial fibrillation was noted, with a heart rate of 99 beats per minute and blood pressure of 113/52 mmHg under treatment with bisoprolol. Physical examination revealed bilateral moderate proptosis and an enlarged thyroid gland. Neither orthopnea nor dyspnea on exertion was present. The lungs were clear upon auscultation, and no evidence of jaundice or pedal edema was noted. Initial investigations (Table 1) revealed markedly decreased white blood cell and neutrophil counts. Although the lymphocyte count was decreased, the degree of reduction was not severe. The red blood cell count, platelet count, and liver function exhibited no marked abnormalities. The CRP level was elevated. Thyroid function tests showed an undetectable TSH level, elevated free triiodothyronine (FT3) and FT4 levels, and positive TSH receptor antibody and thyroid-stimulating antibody results. Thyroid ultrasonography performed on day 2 of the admission revealed an enlarged, heterogeneous, and hypervascular thyroid gland (Fig. 1A and 1B). Chest radiography demonstrated cardiomegaly with a cardiothoracic ratio of 59.6%; however, neither pulmonary congestion nor pleural effusion was noted. Additionally, the oral cavity was poorly maintained, and a crusted bite lesion was observed on the right upper lip. Erythema and swelling were noted extending from the right buccal region to the bilateral mandibular areas. Computed tomography revealed soft tissue opacity in the right buccal region (Fig. 1C), suggesting that right buccal cellulitis had developed as a result of dental infection. No pathogenic bacteria were identified in the blood cultures. His signs and symptoms and laboratory tests meet the diagnostic criteria of thyroid storm based on the diagnostic criteria both of the Japan Thyroid Association (JTA) (TS1 [First version]) and the Burch–Wartofsky Point Scale (BWPS) (85 points: temperature [≥40.0 °C]; tachycardia [90-109 beats per minute]; atrial fibrillation [present]; gastrointestinal-hepatic dysfunction [moderate]; central nervous system disturbance [moderate]; and precipitating event [present]) [5, 6]. Although tachycardia may have been masked by bisoprolol use, potentially affecting the BWPS, the diagnosis of thyroid storm was supported by both the Japan Thyroid Association criteria and the BWPS, which identified the case as definite thyroid storm. Other potential causes of agranulocytosis, including concomitant medications and infection, were also considered [7-11]. Nevertheless, enalapril, spironolactone, and rabeprazole were continued throughout hospitalization; notably, the neutrophil count began to recover promptly after thiamazole was discontinued, suggesting that these drugs were unlikely to be responsible. Although right buccal cellulitis was suspected based on clinical and imaging findings, the patient was hemodynamically stable, and blood cultures were negative. Consequently, sepsis was considered unlikely to have contributed to the agranulocytosis. Therefore, thyroid storm occurred due to right buccal cellulitis with agranulocytosis resulting from the adverse effects of thiamazole.

**Table 1.** Laboratory data on day 0

Test	Result	Reference range
White blood cell count	**610/μL**	3500-8000/μL
	**(0.61** **×** **10^9^/L)**	(3.50-8.00 × 10^9^/L)
Neutrophils	**13.1%**	40.0-71.0%
Absolute neutrophil count*^a^*	**80/μL**	1400-5680/μL*^b^*
	**(0.08** **×** **10^9^/L)**	(1.40-5.68 × 10^9^/L)
Monocytes	**11.5%**	4.0-9.0%
Absolute monocyte count*^a^*	**70/μL**	140-720/μL*^b^*
	**(0.07** **×** **10^9^/L)**	(0.14-0.72 × 10^9^/L)
Lymphocytes	**75.4%**	25.0-45.0%
Absolute lymphocyte count*^a^*	**460/μL**	875-3600/μL*^b^*
	**(0.46** **×** **10^9^/L)**	(0.88-3.60 × 10^9^/L)
Eosinophils	0.0%	0-5%
Basophils	0.0%	0-2%
Red blood cell count	**3.21** **×** **10^6^/μL**	3.80-4.80 × 10^6^/μL
	**(3.21** **×** **10^12^/L)**	3.80-4.80 × 10^12^/L
Platelet count	183 × 10^3^/μL	120-400 × 10^3^/μL
	(183 × 10^9^/L)	(120-400 × 10^9^/L)
AST	20 U/L	13-30 U/L
ALT	14 U/L	7-23 U/L
ALP	**141 U/L**	30-113 U/L
GGT	21U/L	9-32 U/L
Total bilirubin	**1.6 mg/dL**	0.4-1.5 mg/dL
	**27.4 μmol/L**	6.84-25.7 μmol/L
CRP	**14.07 mg/dL**	< 0.3 mg/dL
	**(140.7 mg/L)**	(< 3 mg/L)
TSH	**< 0.05 μIU/mL**	0.50-5.00 μIU/mL
	**(< 0.50 mIU/L)**	(0.50-5.00 mIU/L)
FT3	**4.8 pg/mL**	2.3-4.0 pg/mL
	**(7.37 pmol/L)**	(3.5-6.1 pmol/L)
FT4	**2.5 ng/dL**	0.9-1.7 ng/dL
	**(32.2 pmol/L)**	(11.6-21.9 pmol/L)
TRAb*^c^*	**3.9 IU/L**	< 2.0 IU/L
TSAb*^d^*	**556%**	< 110%

Abnormal values are shown in bold font. Values in parentheses are International System of Units (SI).

Abbreviations: ALP, alkaline phosphatase; ALT, alanine transaminase; AST, aspartate aminotransferase; CRP, C-reactive protein; FT3, free triiodothyronine; FT4, free thyroxine; GGT, gamma-glutamyl transpeptidase; TRAb, TSH receptor antibody; TSAb, thyroid-stimulating antibody; TSH, thyroid-stimulating hormone.

^*a*^Absolute counts were calculated as: white blood cell count × differential fraction (%).

^*b*^Reference ranges of absolute counts were approximated using the institutional reference ranges for white blood cell count and differential fractions, as our laboratory provides only percentage-based reference values for leukocyte subsets.

^*c*^Measured using electrochemiluminescence immunoassay (Elecsys, Roche Diagnostics, Mannheim, Germany).

^*d*^Measured using enzyme immunoassay (BIOSENSOR TSAb YAMASA, YAMASA Corporation, Tokyo, Japan).

## Treatment

**Figure 2.**
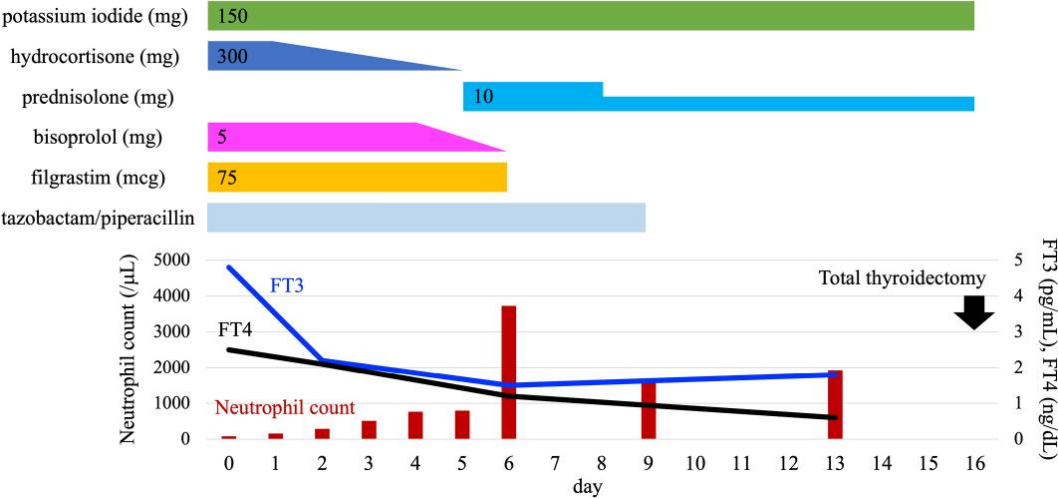
The clinical course of treatment, a neutrophil count, and serum FT3 and FT4 levels. Reference ranges: neutrophil count, 1400-5680/μL (SI: 1.40-5.68 × 10^9^/L); FT3, 2.3-4.0 pg/mL (SI: 3.5-6.1 pmol/L); FT4, 0.9-1.7 ng/dL (SI: 11.6-21.9 pmol/L). Reference ranges of absolute neutrophil counts were approximated using the institutional reference ranges for white blood cell count and differential fractions, as our laboratory provides only percentage-based reference values for leukocyte subsets. Abbreviations: FT3, free triiodothyronine; FT4, free thyroxine.

Treatment was initiated with potassium iodide (150 mg), hydrocortisone (300 mg), and bisoprolol (5 mg) for thyroid storm; filgrastim (75 mcg) for agranulocytosis; and tazobactam/piperacillin for buccal cellulitis (Fig. 2). On day 2, fever and loss of appetite were resolved and thyroid function improved to an FT3 level of 2.2 pg/mL (3.38 pmol/L) and an FT4 level of 2.1 ng/dL (27.0 pmol/L). The dose of hydrocortisone was gradually tapered and changed to 10 mg of prednisolone on day 5, followed by 5 mg on day 8. On day 6, the neutrophil count recovered to 3728/μL (3.728 × 10^9^/L) despite the continuation of enalapril, spironolactone, and rabeprazole, and filgrastim was discontinued. Bisoprolol was discontinued because the patient's pulse rate decreased. On day 8, echocardiography revealed an enlarged left atrium and left ventricular centrifugal hypertrophy; however, the left ventricular ejection fraction was maintained at 64%. On day 9, tazobactam/piperacillin was discontinued because the buccal cellulitis improved. A total thyroidectomy was performed on day 16. Histopathological examination of the excised thyroid gland revealed features consistent with Graves disease.

## Outcome and Follow-up

Postoperatively, potassium iodide and prednisolone were discontinued, and 100 mcg of levothyroxine, 2 mcg of alfacalcidol, and 6 g of calcium lactate were started. On day 190, hypoparathyroidism persisted with a corrected calcium level of 8.5 mg/dL (2.12 mmol/L) (reference range: 8.8-10.2 mg/dL [2.20-2.54 mmol/L]), a phosphorus level of 5.3 mg/dL (1.71 mmol/L) (reference range: 2.5-4.5 mg/dL [0.81-1.45 mmol/L]), and an intact parathyroid hormone level of 9.5 pg/mL (1.01 pmol/L) (reference range: 15.0-65.0 pg/mL [1.59-6.89 pmol/L]). The patient is currently stable on 150 mcg of levothyroxine and 3 mcg of alfacalcidol.

## Discussion

We report a case of agranulocytosis after reinitiating treatment with low-dose thiamazole, which was complicated by the right buccal cellulitis and led to thyroid storm. This case highlights 2 key clinical considerations. Thiamazole can induce agranulocytosis even at low doses and when reinitiated. These issues suggest that the possibility of agranulocytosis should be considered, even in patients who are started on a low dose of thiamazole or are re-treated with it.

Thiamazole can induce agranulocytosis even at low doses. ATD-induced agranulocytosis occurs in 0.1% to 0.15% of cases in Japan, with a median thiamazole dose of 25.2 mg/day at onset and develops within 90 days after the initiation of treatment [12]. In another study in Japan using an administrative claims database, the incidence of leukopenia requiring granulocyte-colony stimulating factor administration in patients newly prescribed antithyroid medication was 0.2% during the first 72 days after initial ATD administration [13]. Recently, among patients who initiated treatment with thiamazole, the incidence of agranulocytosis increased significantly in a dose-dependent manner [14]; nonetheless, no cases of agranulocytosis occurred in patients treated with thiamazole at a dose of 7.5 mg/day or less [14]. ATD-induced agranulocytosis is thought to involve an immune-mediated toxicity and direct toxicity on neutrophils [4, 15]. Recent studies have shown that certain types of human leukocyte antigens (HLA) are associated with susceptibility to ATD-induced agranulocytosis [16, 17]. These findings support the hypothesis that genetically predisposed patients may develop agranulocytosis independent of dosage and that 5 mg of thiamazole can also induce agranulocytosis. Here, HLA typing was not performed because this test is not routinely available or covered by the national health insurance system in Japan. Moreover, the clinical course strongly suggested that thiamazole was the most probable cause of agranulocytosis; other etiologies were considered unlikely.

Thiamazole can also induce agranulocytosis even when reinitiated. The incidence and severity of agranulocytosis did not differ between the initial treatment and reinitiation of the same ATD [13, 18]; however, no cases occurred when ATD was reinitiated within 5 months of discontinuation. These results suggest that a minimum duration of antithyroid drug discontinuation may be required to eliminate inhibitory factors or mechanisms that prevent agranulocytosis [18]. Additionally, a significantly higher proportion of patients who were reinitiated with an alternative ATD and developed agranulocytosis experienced minor adverse effects during the initial ATD treatment [18]. This observation raises the possibility of cross-sensitization or an underlying immunological predisposition, suggesting that clinicians should exercise caution when reinitiating ATD therapy in patients with a history of adverse reactions to either agent. In this case, thiamazole was reinitiated 3 years after discontinuation, and agranulocytosis developed 35 days later. This is consistent with a previous report regarding the interval between the end of the initial treatment course and the start of the subsequent course, as well as the time interval at which agranulocytosis was diagnosed. Conversely, although the initial treatment did not result in even minor adverse effects, agranulocytosis developed after the reinitiation of a minimal dose of an antithyroid drug, specifically 5 mg of thiamazole, leading to agranulocytosis with buccal cellulitis and associated thyroid storm. Although agranulocytosis typically occurs at high doses of thiamazole, the immune-mediated nature of this adverse reaction suggests that even minimal re-exposure can trigger a robust response in sensitized individuals. In this case, although HLA testing was not performed, the rapid onset of agranulocytosis following the reinitiation of low-dose thiamazole may reflect HLA-linked immunological memory reactivation, possibly involving memory T cells. Agranulocytosis was diagnosed by a primary care physician 3 days after the appearance of agranulocytosis symptoms. Adequate patient education regarding potential adverse effects, such as agranulocytosis, is crucial [15], although the patient may not have been informed by the physician when reinitiating ATD.

In conclusion, we report a case of severe agranulocytosis after reinitiating treatment with low-dose thiamazole, which was complicated by right buccal cellulitis leading to thyroid storm. Even in the absence of adverse effects during the initial course of ATD therapy, physicians should remain vigilant for ATD-related adverse effects when reinitiating treatment, even at low doses, particularly after a prolonged discontinuation period. Given the severity of agranulocytosis, patient education at the time of prescription is crucial for the early recognition of symptoms, such as fever or sore throat, which may enable timely diagnosis and appropriate intervention.

## Learning Points

- ATD-induced agranulocytosis is a rare but potentially life-threatening adverse effect that requires prompt recognition and intervention to achieve a favorable prognosis.
- Although typically dose-dependent, agranulocytosis can also occur at low doses of thiamazole.
- Reinitiating thiamazole after prolonged discontinuation may trigger agranulocytosis even in previously tolerant patients.
- Patient education regarding early symptoms, such as fever and sore throat, is essential for timely diagnosis and treatment.

## Data Availability

The original data generated and analyzed during this study are included in this published article.
